# Supplementary material for: Clinical Whole-Genome Sequencing Assay for Rapid Mycobacterium tuberculosis Complex First-Line Drug Susceptibility Testing and Phylogenetic Relatedness Analysis
Source: Microorganisms. 2023 Oct 11;11(10):2538. doi: 10.3390/microorganisms11102538 (PMC10609454; doi:10.3390/microorganisms11102538)
Supplement: Supplementary file 1 [file microorganisms-11-02538-s001.zip › microorganisms-2553049-supplementary.pdf]

microorganisms

MDPI

Article

Clinical Whole-Genome Sequencing Assay for Rapid *Mycobacterium tuberculosis* Complex First-Line Drug Susceptibility Testing and Phylogenetic Relatedness Analysis

Bennett Shaw [1], Benjamin von Bredow [1,2], Allison Tsan [1], Omai Garner [1] and Shangxin Yang [1,*]

[1] Department of Pathology and Laboratory Medicine, UCLA David Geffen School of Medicine, Los Angeles, CA 90095, USA; bmshaw@mednet.ucla.edu (B.S.); benjamin.vonbredow@corewellhealth.org (B.v.B.); attsan@mednet.ucla.edu (A.T.); ogarner@mednet.ucla.edu (O.G.)

[2] Department of Pathology, Oakland University William Beaumont School of Medicine, Rochester, MI 48309, USA

* Correspondence: shangxinyang@mednet.ucla.edu

Table S1. Mutation Summary Table.

Sample ID	RIF	INH	PZA	EMB	STM
UCLA_485			pncA p.His57Asp		
UCLA_487			pncA p.His57Asp		
UCLA_574			pncA p.His57Asp		
UCLA_683		fabG1 c.-15C>T			gid p.Leu79Ser
UCLA_737		katG p.Ser315Thr			
UCLA_789		katG p.Ser315Thr			rpsL p.Lys43Arg
UCLA_798		fabG1 c.-15C>T	pncA c.164_165insGG		rpsL p.Lys88Arg
UCLA_845		fabG1 c.-15C>T			
UCLA_866		S (ahpC p.Asp73His)			gid c.115_115del
UCLA_869		katG p.Ser315Thr			rpsL p.Lys88Arg
UCLA_870			pncA p.His57Asp		
UCLA_1021	rpoB p.Gln432Pro	fabG1 c.-15C>T<br>katG p.Ser315Thr	pncA p.His82Arg	embB p.Met306Ile	
UCLA_1222			pncA p.His57Asp		

Table S2. Growth Media Comparison.

Pair	Sample	Culture Media	MTB AMR WGS Result
1	UCLA 658	Solid	sensitive
	UCLA 659	MGIT	sensitive
2	UCLA 737	Solid	R: isoniazid
	UCLA 738	MGIT	R: isoniazid
3	UCLA 863	Solid	sensitive
	UCLA 864	MGIT	sensitive
